# Clinical significance of small extracellular vesicles in cholangiocarcinoma

**DOI:** 10.3389/fonc.2024.1334592

**Published:** 2024-04-11

**Authors:** Jianjun Wang, Ruizi Shi, Yuan Yin, Hua Luo, Yuan Cao, Yun Lyu, Huiwen Luo, Xintao Zeng, Decai Wang

**Affiliations:** ^1^ Department of Hepatobiliary Surgery, Mianyang Central Hospital, School of Medicine, University of Electronic Science and Technology of China, Mianyang, China; ^2^ National Health Commission (NHC) Key Laboratory of Nuclear Technology Medical Transformation, Mianyang Central Hospital, School of Medicine, University of Electronic Science and Technology of China, Mianyang, China; ^3^ Department of Urology, Mianyang Central Hospital, School of Medicine, University of Electronic Science and Technology of China, Mianyang, China; ^4^ Departmant of Oncology, Mianyang Central Hospital, School of Medicine, University of Electronic Science and Technology of China, Mianyang, China

**Keywords:** extracellular vesicles, exosome, microparticle, cholangiocarcinoma, biomarker, liquid biopsy

## Abstract

Cholangiocarcinoma is an aggressive and heterogeneous malignancy originating from the bile duct epithelium. It is associated with poor prognosis and high mortality. The global incidence of cholangiocarcinoma is rising, and there is an urgent need for effective early diagnosis and treatment strategies to reduce the burden of this devastating tumor. Small extracellular vesicles, including exosomes and microparticles, are nanoscale vesicles formed by membranes that are released both normally and pathologically from cells, mediating the intercellular transfer of substances and information. Recent studies have demonstrated the involvement of small extracellular vesicles in numerous biological processes, as well as the proliferation, invasion, and metastasis of tumor cells. The present review summarizes the tumorigenic roles of small extracellular vesicles in the cholangiocarcinoma microenvironment. Owing to their unique composition, accessibility, and stability in biological fluids, small extracellular vesicles have emerged as ideal biomarkers for use in liquid biopsies for diagnosing and outcome prediction of cholangiocarcinoma. Specific tissue tropism, theoretical biocompatibility, low clearance, and strong biological barrier penetration of small extracellular vesicles make them suitable drug carriers for cancer therapy. Furthermore, the potential value of small extracellular vesicle-based therapies for cholangiocarcinoma is also reviewed.

## Background

Cholangiocarcinoma (CCA) is a highly lethal epithelial tumor accounting for 3% of gastrointestinal malignancies (1, 2). These tumors are heterogeneous and can be divided into three main anatomical subtypes: intrahepatic CCA (iCCA), distal CCA (dCCA), and perihilar CCA (pCCA). Currently, the diagnosis of CCA relies mainly on serum tumor biomarkers, radiographic imaging, and pathological results. However, owing to the insufficient sensitivity and specificity of these diagnostic methods as well as the highly malignant, invasive, and initially insidious nature of CCA, most patients are in the middle to late stages of the disease at the time of diagnosis. The 5-year patient survival after diagnosis has stalled at 10% (3, 4). Although surgery aimed at margin-negative (R0) resection is a potentially curative option for select patients with CCA, the tumors tend to recur and metastasize even after R0 resection. Systemic therapy has become the preferred treatment option for patients unsuited to radical surgery, but its effectiveness is extremely low (5, 6). Advances in genetic profiling suggest that emerging treatment modalities, including immunotherapy and targeted therapy, may prolong the survival of CCA patients; however, most of the emerging treatments are still in clinical trials. Thus, the exploration of new strategies for the diagnosis and treatment of CCA is of significant importance.

Extracellular vesicles (EVs) are nanoscale structures derived from membranes that are released under normal and pathological conditions from various cell types investigated thus far (7). The involvement of EVs in a variety of physiological and pathological activities has been reported, where they are responsible for the transfer of substances and information between parental and recipient cells (8). Based on their formation pathways and sizes, EVs can be classified into three subsets, namely, exosomes, apoptotic bodies, and microparticles (MPs) (**Table 1**). EVs have been found in a variety of biological fluids, including blood, sputum, urine, breast milk, pleural effusions and bronchoalveolar lavage, bile, and ascites fluid (9). In this review, we focus on the first two types of small extracellular vesicles (sEVs): exosomes and MPs. Exosomes are complex structures of approximately 10–100 nm in size, resulting from fusion of multivesicular bodies (MVBs) with the plasma membrane. In constrast, MPs are released outside the cell in a sprouting manner when the cytoskeleton changes during cell activation, with a diameter of about 100–1,000 nm (10, 11). In the tumor microenvironment (TME), sEVs can mediate signal transduction, influencing the proliferation, invasion, and metastasis of tumor cells. It has been reported that these vesicles can transfer surface receptors and deliver bioactive substances (such as proteins, RNAs, and organelles) from one cell to another, which may facilitate intercellular communication and promote extracellular matrix invasion and evasion of immune responses (12, 13). Several studies have suggested the potential advantages of using EVs as biomarkers for non-invasive diagnosis and prognostic prediction (10, 11). Specific tissue tropism, theoretical biocompatibility, low clearance, and strong biological barrier penetration of sEVs make them suitable drug carriers for cancer therapy.

**Table 1:** Classification of sEVs.

Subtypes of sEVs	Exosomes	Microparticles
Other name (s)	Extracellular vesicles	Microvesicles; Ectosomes
Origin	Endosomal membrane	Plasma membrane
Size	10 – 100 nm	100 – 1000 nm
Density	1.13 – 1.19 g/cm^3^	1.04 – 1.07 g/cm^3^
Sedimentation	≥ 100000 g	10000 g
Detection	TEM; Cryo-EM; AFM; NTA; FCM.	TEM; Cryo-EM; AFM; NTA; FCM.
Zeta potential	− 16.35 ~ − 11.85 mV	− 30 ~ − 10 mV
Appearance	Cup-shaped	Irregular-shaped
Mechanisms of the biogenesis	1.ESCRT-dependent mechanism; 2.Synthesis of ceramide that induces vesicle curvature and budding.	1.Increase in cytosolic calcium concentration; 2.Apoptosis-dependent microparticle formation mechanism.
Annexin V binding capacity	No/Low	High
Markers	TSG101, tetraspanins (CD81, CD9, and CD63), and HSP90β	Annexin A1

sEVs: small extracellular vesicles, TEM: transmission electron microscopy, Cryo-EM: cryo-electron microscopy, AFM: atomic force microscopy, NTA: nanoparticle tracking analysis, FCM: flow cytometry, ESCRT: endosomal sorting complexes required for transport, TSG101: tumor susceptibility gene 101; HSP90β: heat shock protein 90β.

In this article, we systematically summarize the biogenesis and release of the two major sEV types, comparing their methods of isolation and purification. The functions of these sEVs in CCA are also discussed. Based on existing studies, the potential clinical application of sEVs in the diagnosing, treating, and prognostic prediction of CCA have also been highlighted.

## Biogenesis, release, and uptake of sEVs

### Exosome biogenesis, release, and uptake

**Figure 1:**
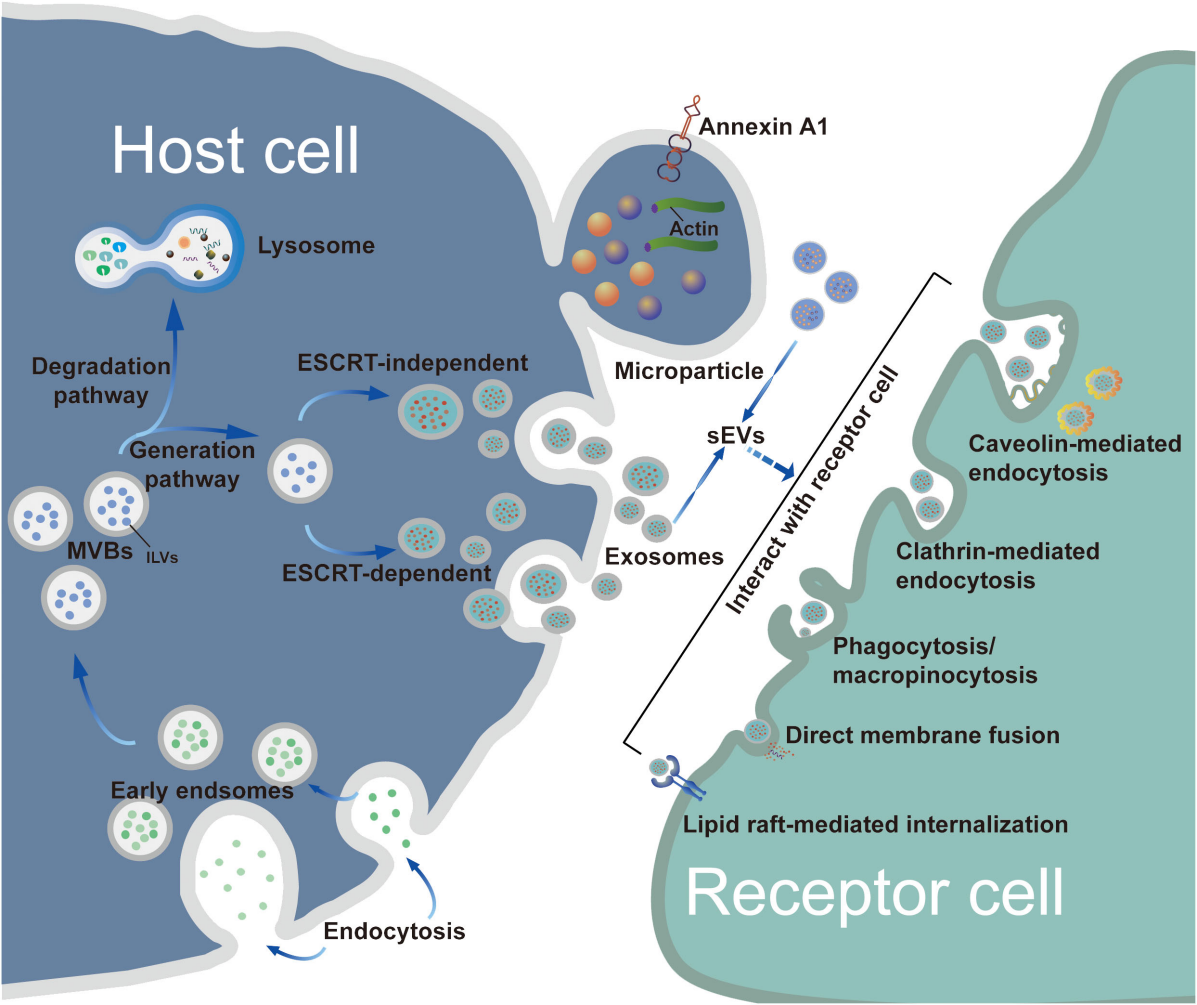
Biogenesis, release and uptake of sEVs. The biogenesis of exosomes consists of three main phases: i) plasma membrane invagination and early endosome formation. ii) generation of ILVs and MVBs. iii) the fusion of MVBs and plasma membrane leads to exosomes release. Generally, the synthesis of MVBs mainly depends on two pathways: ESCRT-dependent and ESCRT-independent mechanisms, with the former being the best characterized pathway. However, MPs originate by outward budding at the plasma membrane. Although the biogenesis of exosomes and MPs are different, the processes by which exosomes and MPs are uptaken by recipient cells are similar. These sEVs can be taken up by target cells through a variety of pathways, such as endocytosis, plasma membrane interactions, and specific protein interactions. As extracellular structures encapsulated in lipid bilayers, sEVs participate in the transportation of bioactive molecules and exchange of information between donor cells and recipient target cells.

Exosome biogenesis includes three main phases: (i) invagination of the plasma membrane and the formation of early endosomes; (ii) generation of intraluminal vesicles (ILVs) and MVBs; and (iii) fusion of MVBs with the plasma membrane resulting in exosome release (14). In general, MVB synthesis is dependent on two pathways, namely, pathways dependent on the endosomal sorting complex required for transport (ESCRT) and ESCRT-independent mechanisms, of which the former is the better characterized (15). The first step in ESCRT-dependent biogenesis is the ubiquitination of membrane proteins of late endosomes by ESCRT-0. This is followed by invagination of the membrane into the MVB lumen triggered by ESCRT-I/II, after which a spiral structure formed with ESCRT-III constricts the neck of the budding vesicle and the final cleavage from the membrane is driven by ATPase VPS4 (16, 17). Once mature, MVBs bind to autophagosomes and are then degraded via the lysosomal pathway or secreted outside the cell as exosomes following plasma membrane fusion (8). The transport of the released MBVs appears to be dependent on the cell type, shown by the ability of RABs to modulate docking of MVBs with the plasma membrane (18, 19). For instance, RAB27 has been shown to mediate MVB docking in several cancer cell lines, whereas RAB35 controls this process only in oligodendroglial cells (20). After being transported and attached to the plasma membrane, MVBs bind to soluble N-ethylmaleimide-sensitive component attachment protein receptors (SNAREs) on the membrane. This process involves small GTPases such as RAL-1 (11). Following MVB fusion with the membrane, the exosomes are released into the extracellular environment. An alternative pathway of exosome biogenesis is associated with ceramide synthesis, inducing vesicle curvature and outgrowth. This pathway depends on raft-based microdomains that segregate cargoes laterally within endosomal membranes and is independent of the ESCRT-dependent mechanism (**Figure 1**). These microdomains are associated with high levels of ceramide precursors and sphingolipids that promote the merging of smaller microdomains, increasing the domain size and inducing outgrowth (21).

After being secreted by host cells, exosomes are taken up by target cells through a variety of pathways, such as endocytosis or interactions with the plasma membrane and specific proteins interactions, with endocytosis being the most widely studied pathway (22). In terms of the components involved, endocytosis can be classified into various types, including phagocytosis, micropinocytosis, and endocytic processes mediated by clathrin, caveolin, and lipid rafts (23). Various proteins, such as integrins, tetraspanins, and lectins, are also associated with exosome uptake, mediated by specific receptor-ligand interactions (14). Due to the heterogeneity of exosomes, it remains controversial whether their uptake is specific (23); therefore, the detailed pathways involved in exosome uptake require further study.

### MPs biogenesis, release, and uptake

Several mechanistic understandings of MP formation have been presented to date; however, a detailed mechanism is not available. Generally, MPs are formed by budding of the plasma membrane. Here, we review the two most widely accepted mechanisms, namely, cell activation and apoptosis (24). When cells are exposed to stimuli (e.g., injury, hypoxia, or ultraviolet radiation), the endoplasmic reticulum releases calcium, resulting in the activation of calcium-dependent enzymes (including scramblases and floppases). This process causes the cytoskeleton to change shape, leading to the transfer of phosphatidylserine to the surface of the cell membrane (25). Furthermore, aggregation of transmembrane proteins and alterations in lipid compositions can affect the curvature of the membrane, resulting in asymmetry of the lipid components (26). This results in shrinkage of actomyosin, outward splitting of the membrane, and release of MPs from the cell. During apoptosis, DNA fragmentation and cell contraction leads to membrane blebbing and MP formation, a process that can last for several hours (27). To date, the detailed mechanisms underlying the formation of MPs remain unclear, thus the need to understand the process involved in MP synthesis.

Although the biogenesis mechanisms of exosomes and MPs differ, the internalization of both exosomes and MPs by cells is similar. The process involves the fusion of sEVs to the membrane of the recipient cell; this may occur by direct fusion with either the plasma or endosomal membranes after endocytosis followed by. Uptake of sEVs occurs via multiple endocytic pathways, as mentioned in the previous section (23). The different components of these mechanisms are targets for blocking the sEV uptake process, thus blocking the oncogenic actions of sEVs derived from tumor cells; these mechanisms include the use of inhibitors of glycosphingolipid synthesis and agents for reducing cholesterol (28–32).

Numerous studies have shown that the biogenesis and release of MPs are influenced by various factors (33–35). For example, ADP-ribosylation factor 1 (ARF1) and ARF6, subfamily members of the G protein, activate downstream signaling. ARF1 can activate RhoA and RhoC, which are involved in the phosphorylation of the myosin light chain (MLC), thus promoting the contraction of actomyosin. RhoA can activate RHO-associated protein kinase (ROCK) and Lim kinase (LIMK), leading to phosphorylation and the prevention of actin cleavage (36). Furthermore, ARF6 can activate RhoA and also phospholipase D (PLD), which directly phosphorylates MLC or recruits extracellular signal-regulated kinase (ERK), resulting in the activation of MLC kinase (MLCK) and the formation of MPs (37). Moreover, some proteins or small-molecule compounds may inhibit MP formation and release. For example, blebbistatin blocks actin filament motility and decreases MP release (38). Similarly, a cytoskeletal regulator, diaphanous-related formin-3, regulates the activation of cofilin, thereby inhibiting membrane outgrowth and MP release (39). In addition, several cell membrane receptors have been implicated in the biogenesis of MPs. P2X7, a purinergic receptor, promotes the release of MPs via the p38 mitogen-activated protein kinase (MAPK) and nuclear factor kappa-B (NF-κB) pathways (40, 41). Others, including tissue factor and α-2-macroglobulin receptor 1, also influence the biogenesis and secretion of MPs (42–44). Environmental and biochemical stimuli participate in the formation and secretion of MPs in different ways.

## Isolation and identification of sEVs

Currently, a variety of sEV isolation and purification techniques have been reported, including differential centrifugation, size-exclusion chromatography (SEC), immunoaffinity capture, ultrafiltration, and microfluidic techniques (45–47). All these techniques can be used for the isolation of sEVs, and each has its own unique advantages and disadvantages (**Table 2**). However, There is a lack of consensus on the methods used by different research teams to isolate and identify sEVs. Isolation and purification of sEVs is usually performed using differential centrifugation, involving the removal of cells and cell debris at low speeds (300–1,000 g) and collection of sEVs at higher speeds (10,000 ×g for MPs and 100,000 ×g for exosomes) (46, 48, 49). It is simple and inexpensive to perform, and allows the separation of particles with varying diameters at different speeds. Differential centrifugation can effectively separate the protein and RNA contents of exosomes and MPs (50–52). However, differential centrifugation cannot distinguish lipoproteins or protein aggregates that are similar in size to sEVs, leading to the question of the accuracy of this method (53). Thus, this method is frequently used in conjunction with methods such as density gradient centrifugation to enhance sEV purity and recovery. SEC is based purely on the size of the particles, and is effective for low volumes where it is capable of separating sEVs from soluble proteins. However, the accuracy of SEC is questionable because particles within the size range of sEVs can be isolated simultaneously. Immunoaffinity capture is effective for the purification of sEV subpopulations but its accuracy is dependent on the selection of suitable affinity reagents and ligand densities for the various sEV types. Ultrafiltration is more suitable for large-scale and repetitive production, and has the advantages of being cost-effective and simple to use (54). Combinations of techniques can help improve accuracy (48, 55). For example, immunoaffinity capture often serves as a further purification method after the isolation of sEVs by centrifugation. The choice of isolation and purification methods depends on the purpose of the study. For large-scale production, guaranteed reproducibility and stability of sEVs is essential. In summary, the current techniques for sEV isolation and purification need improvement, or they will limit the exploration of sEVs in diseases.

**Table 2:** Isolation methods of sEVs.

Method	Mechanism	Advantages	Disadvantages
Centrifugation	Removing cells and debris at a low centrifugation speed and followed by sEVs collection at a higher centrifugation speed.	The most common and efficient isolation method; Low cost; Simple to operate; Not easily contaminated; Ideal for large scale preparations.	High equipment requirements; Time consuming; Risk of aggregation and deformation of sEVs; Risk of protein aggregates.
Size exclusion chromatography	Using a column packed with porous gels, large sEVs flow out fast than small sEVs. The component is separated according to size.	Relatively high purification; Maintaining the biological activity of sEVs; Reducing aggregation of sEVs and proteins.	Time consuming; Not suitable for large scale preparations.
Immunoaffinity	Separated by the specific interaction of antigens on the surface of sEVs and immobilized antibodies.	High purification; Simple to use; Separated sEVs of a specific source.	High-cost antibodies; Not suitable for large scale preparations; Risk of damaging native sEVs structure.
Ultrafiltration	Using specific aperture membranes to remove other components, and retain and concentrate the sEVs.	Separated sEVs of a specific source; Simple to operate; Ideal for large scale preparations.	Low selectivity; Risk of deformation or rupture of sEVs; Possible loss for clogging and membrane trapping.
Microfluidics	The separation is based on several factors, including shape, size, density, electric charge, specific lipid or proteins on sEVs membrane.	High purification; Simple to operate; Capacity of separating and quantifying specific sub-population of sEVs and can be used for diagnosis.	Low sample capacity; Risk of deformation on account of the shear stress.

The International Society of Extracellular Vesicles recommends the identification of EVs according to three aspects, namely, specific morphology, size, and biomarker detection (56, 57). Transmission electron microscopy (TEM), cryoelectron microscopy (cryo-EM), and atomic force microscopy (AFM) are effective for directly evaluating the morphology and size of sEVs (58). Nanoparticle tracking analysis (NTA) based on the Brownian motion principle is useful for analyzing sEV size distributions and concentrations, and can provide information of precise sizes in monodispersed samples although the accuracy can be limited (59). Furthermore, enzyme-linked immunosorbent assays (ELISAs), flow cytometry (FCM), and western blotting (WB) are useful for the detection of specific biomarkers associated with sEVs (58, 60). Considering the advantages and disadvantages of these techniques, combinations of TEM, NTA, FCM, and WB are frequently used for the reliable identification of sEVs. Initially, some transmembrane proteins such as CD81, CD63, and CD9 were reported as representative exosome biomarkers; however, the presence of these proteins has also been reported in MPs and apoptotic bodies (51, 61, 62). In addition, other components associated with exosome formation, such as apoptosis-linked gene 2 (ALG-2)-interacting protein X (Alix), heat shock protein 90β (HSP90β), and tumor susceptibility gene 101 (TSG101) have also been shown to be classical exosomal hallmarks (63, 64). A recent study by Jeppesen et al. identified annexin A1 as a specific MP marker distinct from exosomes (65).

## The roles of sEVs in CCA

Many investigations have demonstrated that cancer progression and metastasis are modulated by alterations in tumor-associated genes as well as the TME (66, 67). The TME contains different types of interacting cells that communicate to promote tumorigenesis. Recently, research on tumor-derived sEVs (TEVs) has received significant attention. As cell-cell communication in the TME influences tumor progression and metastasis, sEVs play key parts in these interactions (68, 69). sEVs are known to be involved in a variety of pathological processes, including the enhancement of invasion and metastasis (70, 71), angiogenesis (72, 73), and tumorigenesis in epithelial cells (74). RNAs and proteins are the most studied components of sEVs; however, a comprehensive summary of their roles in CCA remains insufficient. Most of the data on sEV in CCA comes from data collected in preclinical models, and these reports are mostly exploratory and lack external validity. Therefore, more clinical trials are needed to verify the exact therapeutic effect of sEVs in CCA.

### RNAs of sEVs

The critical role of sEVs in the TME has been extensively studied. MicroRNAs (miRNAs) are documented to be closely involved in CCA. Using miRNA profiling analysis, a study detected several dysregulated exosomal miRNAs between CCA cell lines (KKU-100, KKU-M213, and HuCCA-1) and the normal human cholangiocyte cell line (H69) (75). Significant upregulation of members of the hsa-miR-205-5p and miR-200 miRNA families, including hsa-miR-200c-3p, hsa-miR-200b-3p, hsa-miR-141-3p, hsa-miR-200a-3p, and hsa-miR-429, were observed, whereas members of the miR-199 family, including hsa-miR-199a-5p and hsa-miR-199b-3p, and their clustered miRNA, hsa-miR-214-3p, were markedly downregulated (75). KEGG enrichment analysis indicated that the target genes of these miRNAs were enriched in well-known tumorigenic pathways such as the MAPK and Wnt pathways (75). Eventually, the knockdown of the most upregulated miRNA, miR-205-5p, inhibited CCA cell invasion and metastasis, confirming its role in CCA progression. Moreover, Qin et al. revealed that downregulation of miR-34c in CCA cell-derived exosomes activated cancer-associated fibroblasts (CAFs) by promoting Wnt signaling, ultimately leading to CCA progression (76). Haga et al. examined the role of EV-mediated intercellular signaling in mesenchymal stem cells (MSCs) and CCA cells. They found that CCA-derived EVs enhanced the migratory capability of MSCs as well as alpha-smooth muscle actin (α-SMA) mRNA levels, which contributes to tumor extracellular matrix formation and ultimately promotes CCA development (77). Another study suggested that TEVs delivered miR-210 to CCA cells, where miR-210 specifically decreased reversion-inducing cysteine-rich protein with kazal motif (RECK) expression, which ultimately facilitated growth, metastasis, and chemoresistance in CCA (78).

In addition to miRNAs, other exosomal non-coding RNAs (ncRNAs) are involved in the progression and metastasis of CCA. Wang et al. demonstrated that circ-0000284 levels were increased in exosomes from CCA cell lines in comparison with normal bile duct cells (79). High circ-0000284 expression was found to enhance migration, invasion, and proliferation of CCA cells both in culture and in mouse xenograft models (79). Recently, another similar study reported that the exosomal-associated long non-coding RNA (lncRNA) HCG18 modulates CCA growth and metastasis through the miR-424-5p/SOX9 axis and the PI3K/AKT pathway, with miR-424-5p inhibition reversing the suppression of CCA induced by HCG18 knockdown (80). Interestingly, some sEV ncRNAs have been found to suppress tumorigenesis in the CCA TME. Induction of the epithelial-mesenchymal transition (EMT) by transforming growth factor-β (TGF-β) is involved in CCA invasion and metastasis (81). However, Yu et al. found that transfer of miR-30e by EVs blocked the EMT through direct targeting of Snail, an EMT-induced transcription factor that ultimately suppresses CCA invasion and migration (82). It was also reported by Li et al. that miR-195 overexpression in stromal cells inhibited growth and invasion of adjacent CCA cells. Further research revealed that EVs carrying miR-195 reduced CCA cell growth and enhanced survival in a rat CCA model (83).

sEVs are also involved in regulating tumor progression by participating in immune regulation (84–86). Luo et al. found that exosomes derived from tumor cells carrying miR-183-5p increased the numbers of PD-L1-expressing macrophages, inducing both immune suppression and tumorigenesis in iCCA by activating the miR-183-5p/PTEN/AKT/PD-L1 axis (87). Their results indicate that exosome-associated miR-183-5p may represent a biomarker for the prediction of iCCA progression as well as a possible therapeutic target for iCCA (87). Another study implicated tumor-derived exosomes in CCA immune escape (88). Chen et al. reported the ability of exosomes from the human CCA cell line RBE to block the anti-tumor actions of cytokine-induced killer (CIK) cells through reducing the numbers of CD3(+), CD8(+), NK (CD56[+]), and CD3(+)CD56(+) cells, as well as the production of tumor necrosis factor-α (TNF-α) and perforin (88).

Bile-derived sEVs have attracted the attention of several researchers. Bile is secreted through the biliary ducts into the intestine from the liver. It contains water, biliary acid salts, bilirubin, proteins, electrolytes, mucus, and EVs (89, 90). Pan et al. reported that exosomal miR-200s, particularly miR-200c-3p, found in bile are effective biomarkers for early CCA detection (91). Increased bile contents of exosomal miR-200a-3p and miR-200c-3p and serum contents of exosomal miR-200c-3p were indicative of poor clinical outcome (91). Moreover, Xu et al. reported the transfer of circular-CCA-associated circRNA 1 (CCAC1) associated with bile EVs derived from CCA to endothelial monolayer cells, resulting in disruption of the endothelial barrier and the induction of angiogenesis, promoting both CCA tumorigenesis and metastasis (92).

### Proteins of sEVs

In addition to RNAs, proteins in sEVs are also implicated in the progression of malignant tumors, including CCA (63, 93–95). Zhang et al. demonstrated that exosomal HSPC111 from colorectal cancer (CRC) cells promoted the formation of pre-metastatic niches and liver metastasis by altering lipid metabolism in CAFs, suggesting that HSPC111 may represent a potential therapeutic target (96). Moreover, exosome-associated angiopoietin-like protein 1 (ANGPTL1) reduced CRC metastasis and blocked vascular leakage by the reprogramming of Kupffer cells and reducing the levels of matrix metalloprotein 9 (MMP9) (97). In the CCA microenvironment, the CCA-derived exosomal frizzled class receptor 10 (FZD 10) protein, a family of receptors in the Wnt signaling pathway, may be a potential messenger for cancer reactivation and play an active role in long-distance metastasis (98). Liu et al. observed that low levels of exosomal liver kinase B1 (LKB1) in the plasma were linked with poor outcomes in iCCA patients. In addition, they found that LKB1 could inhibit the immune checkpoint PD-L1 and the metastasis of iCCA cells *in vitro*. These findings suggest new directions for diagnosing and treating iCCA (99). Moreover, proteomic analysis of sEVs from CCA cells revealed multiple CCA-related proteins, such as lactadherin and vitronectin, which are not present in normal bile duct cell exosomes. All these exosomal proteins can induce CCA cell migration and invasion by upregulation of β-catenin (100).

In summary, these findings highlight the important roles of sEVs in the CCA microenvironment and provide further insight into the mechanisms controlling tumorigenesis and metastasis.

## sEVs serve as biomarkers in CCA diagnosis and prognosis

Owing to the stealthiness and heterogeneity of CCA, most patients are unsuited for surgical resection due to the presence of advanced disease, leading to poor prognosis (101). Markers such as carcinoembryonic antigen (CEA) and carbohydrate 19-9 (CA19-9) do not allow for early diagnosis because of their insufficient sensitivity and specificity; these biomarkers can be affected by bacterial cholangitis and cholestasis, together with other factors (102, 103). Liquid biopsy is an emerging diagnostic method that has attracted increasing attention and is expected to compensate for the deficiencies associated with conventional diagnostic methods (104). sEVs carry specific molecules that can be identified in different body fluids and thus have potential for use in the early diagnosis and prognostic prediction of CCA (**Table 3**). However, most of the literature on sEVs as a biomarker for the diagnosis and prognosis of CCA is still immature, and they are mostly preclinical studies. Therefore, it would be worthwhile to explore the potential use of sEVs in response assessment of applied therapies in future studies.

**Table 3:** sEVs as non-invasive biomarkers for diagnosis and prognosis of CCA.

Biomarker	Molecular type	Types of sEVs	Sample	Isolation methods	Case group	Control group	Diagnosis/Prognosis	References
hsa-miR-205-5p↑ miR-200 family members↑	miRNA	Exosomes	Serum	Ultrafiltration&Size exclusion	CCA patients (n = 36)	Healthy individuals (n = 12)	Diagnosis and prognosis	(75)
hsa-miR-214-3p↓	miRNA	Exosomes	Serum	Ultrafiltration&Size exclusion	CCA patients (n = 36)	Healthy individuals (n = 12)	Diagnosis and prognosis	(75)
miR-34c↓	miRNA	Exosomes	Serum	Differential centrifugation & Size exclusion	Human CCA cell lines (HuCCT-1 and QBC939)	Normal human cholangiocyte cell line (HIBEC)	Diagnosis	(76)
miR-210↑	miRNA	EVs	Serum	Not mentioned	Human CCA cell lines (RBE, TFK-1, QBC939, and HuCCT1)	Normal bile duct epithelial cell line (HIBEC)	Diagnosis	(78)
circ-0000284↑	circRNA	Exosomes	Tumor tissues, Serum	TRIzol	Human CCA cell lines (TFK-1, SNU-869, SSP-25, RBE, HuCCT1 and HuH28)	Normal bile duct epithelial cell line (H69)	Diagnosis and prognosis	(79)
circ-CCAC1↑	circRNA	EVs	CCA tissues, Bile	Differential centrifugation & Size exclusion	CCA cells	HUVECs	Diagnosis and prognosis	(92)
HCG18↑	lncRNA	Exosomes	CCA tissues	Differential centrifugation	CCA tumor tissues (n = 60)	Normal tissues	Diagnosis	(80)
miR-30e↓	miRNA	EVs	Serum	Centrifugation&Ultrafiltration	CCA cell lines (HuCCT1, HuH28 and OZ)	Human cholangiocyte cell line (MMNK-1)	Diagnosis	(82)
miR-195↑	miRNA	EVs	Serum	Ultrafiltration	CCA cell lines (LX2, HuCCT, SG231, TFK1, BDEneu, BDEsp)	Normal bile duct epithelial cell line (H69)	Diagnosis	(83)
FZD 10↑	Protein	EVs	Serum	Centrifugation& Ultrafiltration	Human intrahepatic CCA cell line (HUCCT‐1)	Treated CCA cell lines	Diagnosis	(98)
miR-183-5p↑	miRNA	Exosomes	Serum	Not mentioned	iCCA patients (n = 104)	Adjacent non-tumor tissues	Diagnosis	(87)
miR-200c-3p↑	miRNA	Exosomes	Bile	Centrifugation &miRCURY Exosome Kit&Size exclusion	CCA patients (n = 50)	Biliary stone patients (n = 50)	Diagnosis	(91)
Phosphatidylcholine↑	phosphatidylinositol	sEVs	Bile	Ultrafiltration& Size exclusion& Centrifugation	CCA patients (hCCA = 4, dCCA = 3)	Benign diseases patients (gallstones = 6, primary sclerosing cholangitis = 1, autoimmune pancreatitis = 1)	Diagnosis	(105)
CMIP, GAD1, NDPK1, CDS1, CKS1B ↑	mRNA	Exosomes	Serum	Differential centrifugation	CCA patients (n = 12)	PSC patients (n = 6), UC patients (n = 8), and Healthy individuals (n = 9)	Diagnosis	(106)
miR-200c-3p↑	miRNA	Exosomes	Serum	miRCURY Exosome Serum/Plasma Kit	CCA patients (n = 36)	Healthy individuals (n = 12)	Diagnosis and prognosis	(107)
miR-23a-3p↑	miRNA	Exosomes	Tumor tissues	TRIzol	CCA tissues (n = 36)	Adjacent tissues in TCGA database (n =9)	Diagnosis and prognosis	(108)
Cripto-1↑	Polypeptide	Exosomes	Serum	Ultrafiltration&Size exclusion& Centrifugation	pCCA patients (n = 115)	Cholangitis patients (n = 47), and Healthy individuals (n = 65)	Diagnosis and prognosis	(109)
UBE2C, SERPINB1↑	mRNA	EVs	Urine	Differential centrifugation	CCA patients (n = 23)	PSC patients (n = 5), UC patients (n = 12), and Healthy individuals (n = 5)	Diagnosis	(106)
piR-10506469, piR-20548188↑	piRNAs	Exosomes	Serum	Centrifugation & miRNeasy Micro kit	CCA patients (n = 40)	Healthy individuals (n = 50) GBC patients (n = 25)	Diagnosis and prognosis	(110)
HSP90↓	Protein	Exosomes	Serum	Ultrafiltration & Size exclusion& Centrifugation	CCA cell lines (KKU-M213D5)	KKU-M213 cell line	Diagnosis and prognosis	(111)
FGG, A1AG1,S100A8↑	Protein	Exosomes	Serum	Not mentioned	CCA patients (n = 43)	PSC patients (n = 30)	Diagnosis	(112)
Claudin-3↑	Protein	Exosomes	Bile	Ultrafiltration & Size exclusion& Centrifugation	CCA patients (n = 10)	Choledocholithiasis patients (n = 10)	Diagnosis	(113)
AnnexinV+EpCAM+ASGPR1+ CD133+ TMPs↑	TMPs	TMPs	Serum	Not mentioned	Liver cancers (n =172)	Patient with cirrhosis (n = 54)and Healthy individuals (n = 202)	Diagnosis	(114)
LKB1↓	Protein	Exosomes	Serum	Ultrafiltration & Centrifugation	Human CCA cell lines (RBE, HCCC-9810) and plasma of patients with iCCA	The human embryonic kidney fibroblasts 293T	Diagnosis and prognosis	(99)

### Early detection and diagnosis

Several studies aimed at developing sEVs for use in liquid biopsy for CCA have been performed. Existing studies have tended to focus on protein and nucleic acid cargoes, especially miRNAs, showing differential expression between tumor and normal cells, allowing the diagnosis of CCA at an early stage.

A study aimed at the identification of specific non-invasive CCA biomarkers observed differential levels of several RNAs in exosomes from the serum and urine of CCA patients relative to healthy individuals and patients with primary sclerosing cholangitis (PSC) and ulcerative colitis (106). Specifically, the identified RNA transcripts were associated with various tumorigenic processes linked to metabolism, cell-cell communication, energy production, signal transduction, and pathways controlling cell growth and maintenance (106). Five serum biomarkers showed high diagnostic accuracy, including c-Maf-inducing protein, nucleoside diphosphate kinase 1 (NDPK1), glutamate decarboxylase 1 (GAD1), cyclin-dependent kinase regulatory subunit 1 (CKS1B), and CDP-diacylglycerol synthase 1 (CDS1) (106). Their levels in serum exosomes were elevated, with AUC values of 0.957, 0.928, 0.899, 0.893, and 0.891, respectively, for CCA diagnosis compared to controls (106). In addition, the urinary exosomal mRNA cluster consisting of the ubiquitin-binding enzyme E2C (UBE2C) and the serine protease inhibitor B1 (SERPINB1) was increased, suggesting its potential in CCA diagnosis, which requires further evaluation in the future (106). Shen et al. reported that five exosomal miRNAs from CCA peripheral blood samples were significantly upregulated compared to those from healthy individuals (107). Among these, four miRNAs in the miR-200 family (miR-141-3p, miR-200a-3p, miR-200b-3p, and miR-200c-3p) exhibited higher AUCs than CA19-9 (0.78), with miR-200c-3p exhibiting the highest diagnostic power with an AUC of 0.93 (107). Similarly, Ni et al. demonstrated significant upregulation of exosomal miR-23a-3p in both CCA tissues and CCA cell lines (QBC939, RBE, HUCCT1, and HCCC9810) compared with intrahepatic bile duct epithelial cell lines of normal human HiBECs, which provided potential evidence for the early diagnosis of CCA (108). Moreover, a recent study showed elevated levels of serum exosomal Cripto-1 in patients with pCCA, suggesting its potential as a biomarker for diagnosing pCCA (sensitivity: 79.1%; specificity: 87.5%). Moreover, the AUC value of Cripto-1 for pCCA diagnosis (0.874) was much higher than the AUC values of CA19-9, CEA, and the combined AUC values of CA19-9 and CEA (0.773, 0.596, and 0.773, respectively) (109). Some downregulated exosomal miRNAs have a diagnostic value. For example, Kitdumrongthum et al. reported that exosomal miR-214-3p and miR-199 family members were downregulated in CCA cells relative to cholangiocyte lines (75). This suggests that these downregulated exosomal miRNAs may be useful biomarkers for diagnosing CCA (75). In addition to miRNAs, several circulating sEVs and ncRNAs are expected to serve as diagnostic biomarkers for CCA. Gu et al. revealed that some differentially expressed exosomal piwi-interacting RNAs (piRNAs) in the plasma could be useful biomarkers for diagnosing and predicting prognosis in CCA and gallbladder carcinoma (GBC) because of their easy and quick accessibility. For example, the plasma contents of piR-10506469 and piR-20548188 are increased in patients with CCA and GBC, whereas they are significantly downregulated in patients who have undergone surgery (110). Another study showed that circ-0000284 (circHIPK3) levels were marked raised in CCA cells, tissues, and plasma exosomes, indicating its diagnostic value in CCA (79). Considering that the enrichment and stability of sEV-associated circRNAs, they may represent useful non-invasive biomarkers.

Research on sEV proteins has identified several potential targets for the diagnosis of CCA (115, 116). Proteome profiles revealed multiple differentially expressed proteins in the serum exosomes of CCA, PSC, and healthy groups (111). These proteins, including aminopeptidase N (AMPN), polymeric immunoglobulin receptor, and pantetheinase (VNN1), are abundant in CCA serum exosomes compared to controls (111). Similarly, it has also been reported that the fibrinogen gamma chain, S100A8, and alpha1-acid glycoprotein 1 (A1AG1), are consistently overexpressed in EVs from CCA compared with PSC tissues (112). As described above, exosomes with low levels of LKB1 protein induce expression of the immune checkpoint PD-L1, the malignant phenotype of iCCA cells *in vitro*, and cancer metastasis *in vivo* (99).

However, few studies have explored the possibility of using MPs as diagnostic and prognostic indicators of CCA. Henrike et al. evaluated the diagnostic value of tumor-derived MPs (TMPs) in detecting and monitoring hepatocellular carcinoma (HCC) and CCA (114). They demonstrated elevated annexinV+ EpCAM+ CD147+ TMPs in liver cancers, including HCC and CCA. Meanwhile, annexinV+ EpCAM+ ASGPR1+ CD133+ TMPs can distinguish HCC, CCA, and cirrhosis patients from those without tumors (114). These results strongly suggest that annexinV+ EpCAM+ ASGPR1+ TMPs may be effective non-invasive biomarkers for use in HCC and CCA liquid biopsies.

Moreover, sEVs in bile have received considerable attention from researchers. Severino et al. investigated non-invasive biomarkers for CCA, reporting that the estimation of EVs in bile allowed for the correct classification of all malignant and nonmalignant common bile duct (CBD) stenoses (117). Relative to serum CA19-9, bile EV concentrations were found to show markedly greater accuracy in the diagnosis of malignant CBD stenoses. Their findings also suggested that, compared to bile carcinoembryonic cell adhesion molecule 6 (CEAM6), one of the most promising biomarkers for malignant stenosis in bile, EVs are the best biomarkers for malignant stenosis in the CBD (117). Similarly, Ikeda et al. reported the enrichment of Claudin-3 in exosomes from human bile, suggesting its potential as a CCA biomarker, with a diagnostic sensitivity and specificity of 87.5% (113). Muraki et al. emphasized the critical role of phosphatidylcholine in human bile. They found that the phosphatidylcholine levels of sEVs in human bile may be a biomarker for CCA, with a sensitivity of 71.4% and a specificity of 100% (105). In summary, these findings emphasize the potential value of sEVs in the diagnosis of CCA; however, future validation in large cohorts is still necessary.

However, despite the favorable specificity and sensitivity of sEV-mediated early detection and diagnosis of CCA, there are still many problems that need to be resolved before they can be further developed. First, the efficiency of isolating sEVs from body fluids must be increased, and reproducibility must be guaranteed. All existing techniques for the isolation and identification of sEVs have unique advantages and disadvantages, and combinations of these techniques may help improve their accuracy. Large-scale reproducibility and stability need to be considered first. Secondly, the specificity and accuracy of sEV-associated biomarkers and their cargoes require validation in large CCA cohorts. Furthermore, researchers have tended to pay greater attention to exosomes than MPs, which might be a result of the absence of established standard protocols for MP extraction, enrichment, and identification. Thus, the detailed mechanisms involved in the biogenesis, capture, and uptake of MPs must be further elucidated.

### Prognostic value

Compared with their diagnostic value, there has tended to be greater focus on the use of sEVs in predicting the prognosis of CCA. Lapitz et al. found that the levels of exosomal tctex1 domain-containing 2 (TCTEX1D2) were increased in poorly differentiated tumors relative to differentiated tumors, and proposed that TCTEX1D2 may function as a biomarker for the non-invasive prediction of prognosis in CCA (106). Another study observed a positive association between exosomal miR-200a/c-3p and CCA stage. Specifically, serum exosomal miR-200a/c-3p levels were markedly increased in patients with stage III–IV disease in comparison with those with stage I–II CCA (P<0.05) (107). Moreover, Ni et al., exploring the associations between exosomal miR-23a-3p and the clinicopathological characteristics of CCA patients, reported a significant link between exosomal miR-23a-3p expression and lymph node metastasis in both iCCA (P=0.020) and dCCA (P=0.027) (108). Furthermore, Hu et al. found that exosomal Cripto-1 is associated with pCCA metastasis and independently predicted poor survival in patients with pCCA (109). Specifically, the TMN stage and metastasis, both distant and lymph node, in patients with pCCA increase with increasing levels of exosomal Cripto-1 (109). As mentioned in the previous chapter, some piRNAs, such as piR-10506469 and piR-20548188, are differentially expressed before and after surgery and may thus be biomarkers for prognosis prediction in patients with CCA and GBC (110). Wang et al. discovered that high expression of circ-0000284 in CCA cell lines, tumor tissues, and plasma exosomes enhanced the migration, invasion, and proliferation of tumor cells *in vivo* and *in vitro* (79). Another study confirmed differential phosphorylation of HSP90 in invasive CCA cells, suggesting its use as marker for metastatic CCA (111). Meanwhile, Liu et al. found that low plasma levels of exosomal LKB1 were linked with poor outcomes in patients with iCCA (99).

In summary, these results highlight the prognostic value of sEVs in CCA, which may be beneficial for tumor staging, aggressiveness, and risk of recurrence. In the future, sEVs could be used clinically to monitor the response of tumors to treatment. However, most of these studies are still in their early stages, and many follow-up studies are necessary to confirm their authenticity and validity.

## The potential applications of sEVs in CCA treatment

Traditional artificial synthetic drug delivery systems, such as polyethylene glycol (PEG) and liposomes, are useful for drug delivery (118–120). However, the potential immune responses, off-target effects during systemic circulation, and dangerous clinical profiles limit their application (121, 122). sEVs are extracellular structures encapsulated in lipid bilayers, and their initial function is to participate in the transportation of bioactive molecules and the exchange of information between donor and recipient target cells. Several studies have shown that sEVs have some of the characteristics of their parental cells, suggesting their superior biocompatibility relative to artificial drug delivery vehicles, such as lipid-based nanoparticles or liposomes (70, 123, 124). In addition, their natural origin gives them an immunological advantage that can reduce drug clearance and immune responses compared with artificial drug delivery vehicles (125, 126). Therefore, the specific tissue tropism, theoretical biocompatibility, low clearance, and strong biological barrier penetration of sEVs make them suitable drug carriers for cancer therapy.

**Figure 2:**
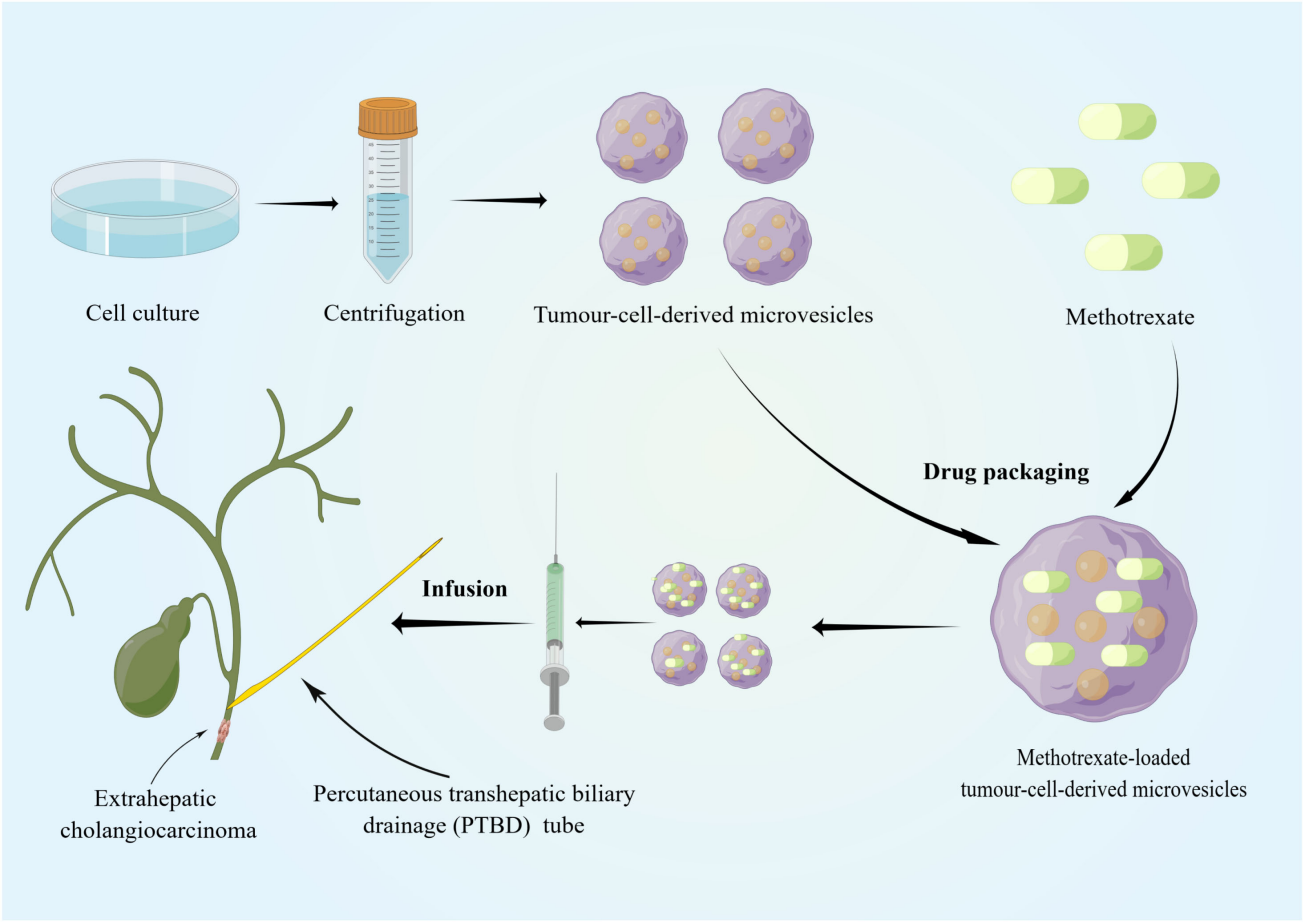
Tumor cell lines and tumor-cell-derived microvesicles have been specially processed to enable them to be combined with conventional chemotherapeutic drugs. The specific tissue tropism, theoretical biocompatibility, low clearance and strong biological barrier penetration of sEVs make them more suitable as drug carriers for cancer therapy than other artificial drug delivery vehicles. MTX-TMPs are injected into the bile duct lumen of dCCA patients through a PTBD tube and then exhibit strong tumor-killing effects *in vivo*. By Figdraw (www.figdraw.com).

Recently, sEVs has been used in the clinical treatment of cancer (127–129), for example, pancreatic cancer (130, 131) and lung cancer with malignant pleural effusion (132). However, the use of sEVs as therapeutic targets for CCA remains in the exploratory stage, and research is currently mainly focused on their role in diagnosis and prognosis. Regarding their therapeutic potential, the use of sEVs in treating CCA was attempted by a Chinese group. Most patients with dCCA develop extrahepatic biliary obstructions, and palliative biliary drainage becomes the standard treatment for these patients (133, 134). However, many problems remain associated with long-term biliary drainage, including drainage tube migration and occlusion, infection, and cholangitis (135). Considering that sEVs can be biological carriers of chemotherapeutic drugs and immune modulators, Gao et al. infused MTX-TMPs into the bile duct lumen of patients with dCCA through a percutaneous transhepatic biliary drainage tube (136) (**Figure 2**). In their study, MTX-TMP perfusion effectively treated obstructive dCCA, relieving biliary obstruction in 25% of patients, with this relief persisting for relatively long period without symptoms of discomfort, such as abdominal pain and vomiting. In their study, a patient showed significant relief of obstruction within 5 months after MTX-TMP perfusion (136). Furthermore, they revealed that MTX-TMPs could use uridine diphosphoglucose and complement the C5 pathways to induce the recruitment of neutrophils to the tumor site and subsequently induce the death of CCA cells. In summary, these findings suggest that MTX-TMPs may be a possible treatment for patients with obstructive dCCA.

Additionally, sEV-mediated CCA immunotherapy may be a promising target for new clinical strategies. Tumor-derived exosomal miR-183-5p promotes immunosuppression and disease progression in iCCA by upregulating macrophages expressing PD-L1 via the miR-183-5p/PTEN/AKT/PD-L1 pathway (87). These results suggest that miR-183-5p may be a candidate target for the treatment of immune tolerance in iCCA. Chen et al. found that exosomal circ-0020256 produced by tumor-associated macrophages promotes the proliferation, migration, and invasion of CCA cells (137). These findings revealed that interfering with circ-0020256-mediated communication between CCA cells and tumor-associated macrophages may be a promising therapeutic strategy.

Blocking the expression of specific miRNAs in sEVs has potential for treating CCA. Ni et al. showed that exosomal miR-23a-3p could promote the proliferation, migration, and invasion of CCA in animal models by interacting with Dynamin3, which demonstrated that miR-23a-3p has the potential to be a therapeutic target for CCA (108). As mentioned previously, several studies have found that other miRNAs in exosomes (e.g., miR-195 and miR-30e) can effectively inhibit the development of CCA, indicating that upregulation of these miRNAs may contribute to reducing the burden of CCA (82, 83). Although these findings strongly support the usefulness of sEVs for treating CCA, their applications in clinical practice remain in the future. sEVs has potential value in the clinical treatment of CCA, but a large number of subsequent clinical studies are still needed to confirm its effectiveness.

## Conclusions and future perspectives

Currently, most patients with CCA are diagnosed when the disease has reached an advanced stage, and radical surgical resection represents the only possible curative treatment. Postoperative recurrence and metastasis lead to a poor prognosis and low 5-year survival rates. Thus, it is essential to identify effect methods for the early diagnosis and treatment of CCA, including the identification of therapeutic targets. sEVs can be obtained from numerous body fluids, and certain nucleic acids or proteins present in them have advantages in terms of abundance and stability, making them promising non-invasive biomarkers. In addition, sEVs have good histocompatibility and can partially reflect the specific characteristics of the parental cells; therefore, the modification of sEVs for CCA therapy has great potential. However, several issues must be addressed when applying sEV-based approaches before they can be further developed. First, the current techniques for isolating and purifying sEVs requires further optimization to improve both efficiency and convenience. Furthermore, the mechanisms of biogenesis, release, and uptake of sEVs, as well as the molecular mechanisms associated with the progression of sEVs in CCA, are not fully understood; hence, exploration of the internal details remains necessary. Finally, the existing artificial synthetic sEV techniques are still immature, and relevant clinical trials are insufficient; thus, the development of sEVs as drug carriers for the treatment of CCA still faces many challenges. In conclusion, research on sEVs is still in its infancy, and further work is necessary on both sEV-associated biomarker identification and therapy. Theoretical and technical bottlenecks require resolution before our understanding of sEVs can be translated into clinical practice.

## Author contributions

JW: Writing – original draft, Writing – review & editing. RS: Methodology, Writing – original draft, Writing – review & editing. YY: Writing – review & editing, Conceptualization, Investigation. HL: Writing – review & editing, Resources, Supervision. YC: Writing – review & editing, Visualization. YL: Writing – review & editing, Methodology, Validation. HWL: Writing – review & editing, Conceptualization. XZ: Writing – review & editing, Supervision. DW: Supervision, Writing – review & editing.
